# Assessment of Vitality, Blood Profile, and Degree of Meconium Staining on the Skin in Newborn Dogs According to Its Birth Weight

**DOI:** 10.3390/vetsci10070453

**Published:** 2023-07-11

**Authors:** Karina Lezama-García, Julio Martínez-Burnes, Uri Baqueiro-Espinosa, Adriana Olmos-Hernández, Ismael Hernández-Ávalos, Adriana Domínguez-Oliva, Daniel Mota-Rojas

**Affiliations:** 1 PhD Program in Biological and Health Sciences, Doctorado en Ciencias Biológicas y de la Salud, Universidad Autónoma Metropolitana, Mexico City 04960, Mexico; 2 Facultad de Medicina Veterinaria y Zootecnia, Universidad Autónoma de Tamaulipas, Victoria City 87000, Mexico; 3 School of Biological Sciences, Queen’s University Belfast, Belfast BT9 5DL, UK; 4 Division of Biotechnology—Bioterio and Experimental Surgery, Instituto Nacional de Rehabilitación-Luis Guillermo Ibarra Ibarra (INR-LGII), Tlalpan, Mexico City 14389, Mexico; 5 Clinical Pharmacology and Veterinary Anesthesia, Facultad de Estudios Superiores Cuautitlán, Universidad Nacional Autónoma de México (UNAM), Cuautitlán Izcalli 54714, Mexico; 6 Neurophysiology, Behavior and Animal Welfare Assessment, DPAA, Universidad Autónoma Metropolitana (UAM), Unidad Xochimilco, Mexico City 04960, Mexico

**Keywords:** whelping, animal perinatology, puppy welfare, newborn puppy, vitality, meconium staining, blood biomarkers

## Abstract

**Simple Summary:**

Preventing neonatal mortality is a critical aspect of canine perinatology. Among the leading causes of mortality, a low vitality score at birth, hypothermia, hypoxia, and hypoglycemia can be mentioned. This study aimed to assess vitality, blood values, and the degree of meconium staining on newborns’ skin concerning their birth weight. It is concluded that larger newborns tend to present greater problems in surviving.

**Abstract:**

Neonatal mortality in dogs reaches up to 40%. Due to the high rates, promptly detecting the causes and preventing newborns from dying are extremely important. Vitality evaluation, blood parameters, and the degree of meconium staining on the skin are valuable resources in canine perinatology. In this study, 435 puppies from 85 bitches close to parturition were recruited and divided into four quartiles according to the puppy’s birth weight: Q_1_ (127–200 g) n = 110 puppies, Q_2_ (201–269 g) n = 108 puppies, Q_3_ (270–388 g) n = 108 puppies, and Q_4_ (389–464 g) n = 109 puppies. This experimental article aimed to report the effect of birth weight on the blood profile variables, the vitality of newborn puppies, and the meconium staining degree, integrating these three aspects. It was concluded that the weight of newborns was correlated with the degree of meconium staining, presenting more cases of severe meconium staining in the puppies of the highest birth weight group. The weight of the newborns was correlated with a higher number of stillbirths and alterations in the blood variables, showing the most severe cases of metabolic acidosis, hypoxia, and hypoglycemia in the puppies of the Q_4_ quartile. On the contrary, no statistically significant correlations were found between the weight of newborns and vitality. Nevertheless, the analysis of the results showed that the most vigorous puppies were found at Q_1;_ however, at minute 60 after birth (AB), all the puppies in the four quartiles standardized their vitality scores.

## 1. Introduction

During whelping, dogs’ mortality rates can reach up to 40% [1]. For this reason, these high rates worry dog owners and breeders [1,2] since one in ten puppies can die before 60 days of age [3]. In addition, newborn puppies are highly immature, making them extremely vulnerable [4,5]. Perinatal death could occur before parturition when the fetus is forming in the uterus, during expulsion, immediately after birth, or during the first weeks of life [2,6,7], especially on the first seven days [8,9], but during birth is when the majority of stillbirths occur [10].

In dogs, similarly to humans, pigs, and cattle [11], puppies with less birth weight are more likely to die. For example, in piglets, this risk is a dozen times more likely than in animals with normal weight at birth [11].

One factor observed to affect newborns’ adaptation to extrauterine life negatively is asphyxia [12,13], including both their vitality and viability. This, in turn, can delay the newborn reaching the dam’s teat [8,14,15]. There have been some reports in dogs that have presented a certain level of transitory asphyxiation during parturition. In a certain way, this can become normal during parturition, generating in puppies transitory acidosis and hypercapnia [16,17,18]. The gas exchange could be altered if these conditions continue [19], decreasing respiration rates and generating metabolic acidosis in newborns [20]. Fortunately, blood gases can be evaluated through the umbilical cord in other species, such as humans. In this way, we can obtain valuable information regarding the neonatal acid–base status [21,22]. Therefore, gasometry is currently an important tool for assessing the health status of newborns [8,23]. In small species, the assessment of umbilical blood parameters is limited. However, it is a technique that has been applied to newborn piglets to evaluate concentrations of pH, partial pressure of oxygen (pO_2_), partial pressure of carbon dioxide (pCO_2_), glucose, lactate, hematocrit, sodium, potassium, and ionized calcium [24].

Newborn mortality risk also includes the proportion of liveborn (LB) vs. stillbirth (SB) puppies and their viability [25,26], with hypoxia being a determinant factor that can alter a newborn’s blood profile [27,28,29].

On the other hand, meconium staining of the skin at birth and aspiration of meconium reflect dystocia processes with severe intrauterine hypoxia [30]. Newborns exposed to meconium aspiration develop Meconium Aspiration Syndrome(MAS) [31]. MAS increases neonatal mortality due to hypoxemia, acidosis, respiratory distress [31,32,33], and pulmonary edema due to the proinflammatory mediators contained in meconium [34]. MAS has been reported to occur in diverse species; for example, in puppies, the reported mortality from MAS can reach 1–3% [35]. Various articles explain the pathophysiology of MAS; however, Swarman et al. [36], Martínez-Burnes et al. [31], and Mota-Rojas et al. [33] describe the association between MAS, airway obstruction, and fetal hypoxia, this being one of the most critical factors that can cause a loss of vitality in newborns. 

Neonatal vitality refers to the capacity of newborns to respond to parturition stress. Vitality is evaluated by the Apgar scoring system adapted for human and animal newborns [2,37]. According to Randall [38], low viability scores in piglets are related to low birth weights and hypercapnia and, as stated by Zaleski and Hacker [39] and De Roth and Downie [40], are also positively associated with pH and negatively with PCO_2_.

Although studies in dogs assessing the effect of the dam’s weight on the puppy’s birth weight, litter size, vitality, survival [1,41,42], and the occurrence of asphyxia and newborn hematological values have been evaluated [27], the effect of the birth weight on the blood profile, meconium staining degree, and vitality has not been comprehensively studied. Therefore, this study aimed to evaluate the effect of birth weight on blood profile variables, the vitality of newborn puppies, and the meconium staining degree, integrating the three aspects. We hypothesized that bigger newborns would have lower vitality scores, significant blood profile alterations, and more cases of meconium staining than smaller puppies.

## 2. Materials and Methods

### 2.1. Facilities 

This study was developed in the facilities of 10 veterinary hospitals in the municipality of Campeche, Campeche State, Mexico, where there is a tropical climate with a temperature between 36 and 40 °C. To carry out this study, the tutors of pregnant bitches were asked for their collaboration. The bitches were given medical attention and monitoring from day 25 of gestation until 48 after the puppy’s birth. All births took place in the clinics. Once the probable date of parturition was calculated, some bitches stayed sheltered in the clinics, or the guardians took them to the clinics when they began to notice changes in the bitches’ behavior. The tutors did not participate during the whelping; only veterinary staff attended and monitored it.

### 2.2. Study Population

Four hundred thirty-five puppies from eighty-five parturient bitches were recruited and divided into four groups classified in quartiles, following Mugnier et al. [43] and Tesi et al.’s [44] method. The first quartile (Q_1_) represents the lowest 25% of registered values, the second quartile (Q_2_) represents 25–50%, the third quartile (Q_3_) represents 50–75%, and the fourth quartile represents 75–100% (Q_4_). Animals in group Q_1_ were considered low-weight, while those belonging to Q_4_ were considered high-weight puppies. This classification is based on the great variety of dog breeds, ranging from Chihuahuas weighing 500 g as adults to mastiffs weighing 100 kg [45]. Within the breeds included in this study, we can mention Chihuahua, Yorkshire Terrier, Poodle, Scottish Terrier, Cocker Spaniel, Standard Schnauzer, German Shepherd, Labrador, Golden Retriever, Great Dane, and Belgian Shepherd. Quartiles were calculated at the puppy level with this formula: Qa = Li ((aN/4 + Fi-1)/Fi) Ai, where Li is the lower limit of the class where the quartile is located, N is the sum of the absolute frequencies, Fi-1 is the accumulated frequency of the previous class, and Ai is the amplitude of the class, that is, the number of values contained in the interval. The groups were Q_1_ (127–200 g) n = 110 puppies, Q_2_ (201–269 g) n = 108 puppies, Q_3_ (270–388 g) n = 108 puppies, and Q_4_ (389–464 g) n = 109 puppies. 

The inclusion criteria were the same used in previous studies by Reyes-Sotelo et al. [27,28] and Lezama-García et al., 2022. Mota-Rojas et al.’s [29,46] classification of type I and type II stillbirths in piglets was used to define which could be considered for the study and which could not. According to Mota-Rojas et al. [30,46], stillbirths can be classified into two types: type I, also known as prepartum or antepartum deaths, includes fetuses that die before the end of gestation, usually of infectious causes and have a rather characteristic hemorrhagic and edematous appearance with a grayish-brown discoloration; type II stillbirths, which are also referred to as intrapartum deaths, can die during parturition typically from intrauterine asphyxia and rarely from infectious diseases, and they have the same appearance of their normal littermates, but they do not breathe. Type I stillbirths were excluded from the study, and only type II stillbirths were included, classified by necropsy. 

Puppies were weighed using a digital scale from Salter Weight Tronix Ltd., West Bromwich, UK, immediately after the dam stopped licking and cleaning their amniotic fluids and placental membranes. 

### 2.3. Blood Sampling

Blood samples were taken by a veterinarian when the bitch finished removing the chorioallantoic membranes. When necessary, an assistant held the puppy in a supine position and exposed the abdominal region. When the puppies allowed it, they were only placed in lateral decubitus on an adult diaper to perform the sampling. Venous blood from the umbilical cord (0.3 mL) was obtained with a tuberculin syringe and a lithium heparin-impregnated needle. All samples (150 µL) were processed using a GEM Premier^®^ critical blood variable analyzer (Instrumentation Laboratory Diagnostics, Lexington, KY, USA/Milano, Italy). The metabolites analyzed were glucose (mg/dL), lactate (mg/dL), blood gases pCO_2_ (mmHg), pO_2_ (mmHg) pH, HCO_3_ − (mmol/L), EB (mEq/L), Ca++ (mmol/L), and hematocrit (Htc %). All profiles were tested for each LB and type II SB pup.

### 2.4. Meconium Staining Degree on Skin

**Figure 1:**
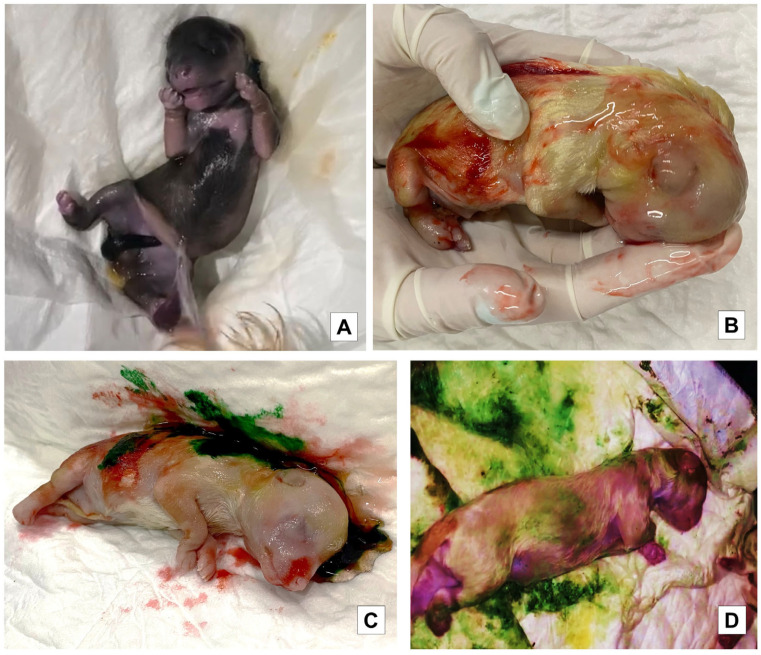
Different degrees of meconium staining on the skin in liveborn (LB) puppies. (**A**). Liveborn (LB) with amniotic fluid and no meconium staining. (**B**). Liveborn (LB) with mild meconium staining. (**C**). Liveborn (LB) with moderate meconium staining. (**D**). Liveborn (LB) with severe meconium staining.

To assess the degree of meconium staining in the skin of the puppies, they were divided according to the methodology of Mota-Rojas et al. [30] into absent, mild, moderate, and severe (Figure 1). It is important to emphasize that the process of resuscitation of the newborns by the mother was not interrupted since the puppies were taken just after the bitch finished removing the chorioallantoic membranes and as soon as possible to prevent the bitch from licking the meconium stain; once the stain was impregnated in the white towel, the puppy was returned to the dam so that she could continue with the licking and maternal care.

**Figure 2:**
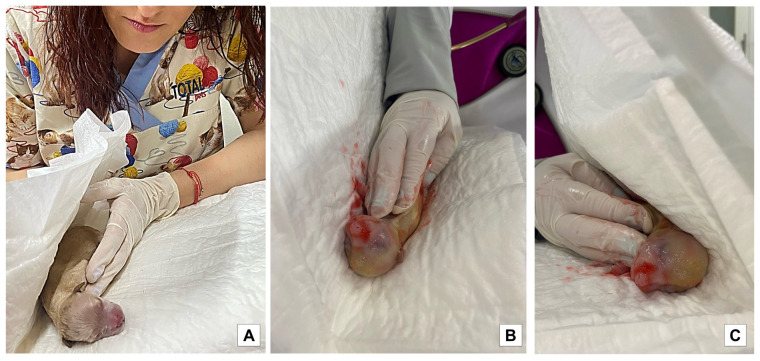
Methodology used to evaluate the degree of meconium staining in puppies. (**A**): The pup was gently picked up before the bitch began to lick it to prevent her from removing meconium staining if it was present. (**B**,**C**): The puppy was placed in a white diaper and surrounded with it to impregnate the meconium staining.

**Figure 3:**
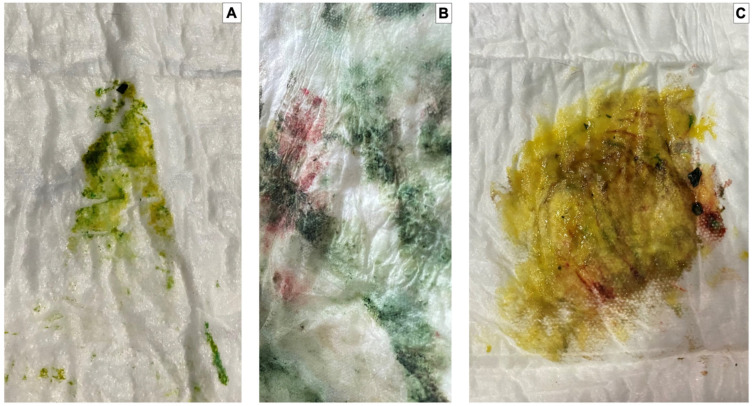
Different degrees of meconium staining. Different degrees of meconium staining were observed using the methodology described in the previous figure. (**A**): Mild. (**B**): Moderate. (**C**): Severe.

The puppies born free of meconium staining on the skin were considered absent of staining, a mild degree was considered when the body was covered less than 30% of its surface, a moderate degree was considered by 30 to 60%, and a severe degree was considered when its body was covered over 60%. A white human bed diaper was used to identify the degree of meconium staining in the dark-colored animals, pressing it on the newborn body to more clearly observe the impregnated color (Figure 2 and Figure 3). It is worth mentioning that the puppies were removed from the mother as soon as possible at birth to prevent the bitch from licking the meconium stain; once the stain was impregnated in the white towel, the puppy was returned to the dam so that she could continue with the licking and maternal care.

### 2.5. Vitality Score

**Figure 4:**
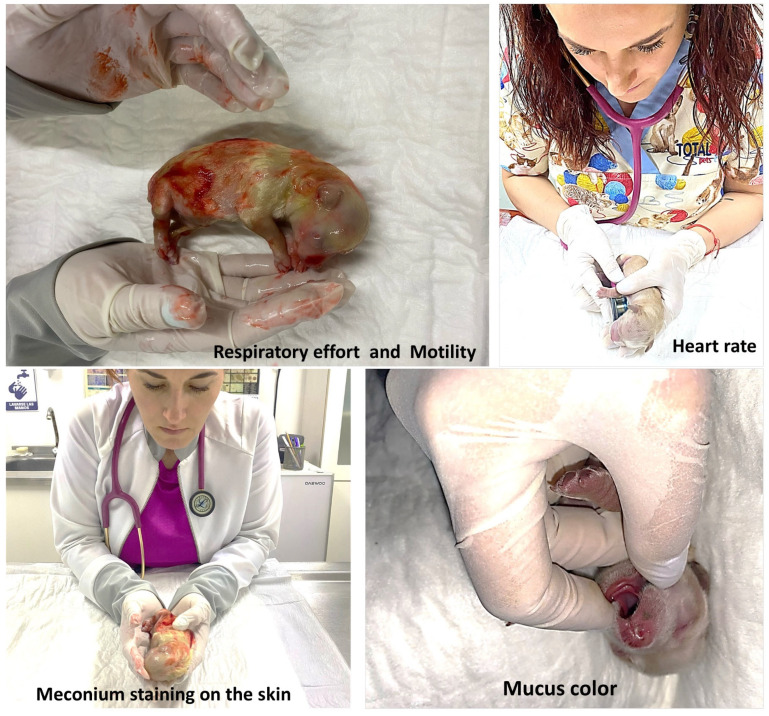
Evaluation of vitality score in first minute after birth.

For the evaluation of vitality in the newborns, the Veronesi [1] scale was used with Randall’s [38] adaptation of the Apgar score for human newborns and modified by Mota-Rojas et al. [47]. The variables measured in the first minute after birth were respiratory effort (no crying/< 6 respiratory rates (rr), mild crying/6 to 15 rr, and crying/>15 rr); motility (flaccid, some flexions, and active motion); heart rate (beats per min): <180, between 180 and 220, and >220; mucus color (cyanotic, pale, and pink). Also, the meconium staining on the skin was classified as severe, moderate, mild, or absent based on the methodology used before in pigs by Mota-Rojas et al. [30,47]. The score for vitality was 0 (the least favorable) to 2 (the most favorable), and a global score ranging from 1 to 10 was obtained for each newborn puppy (Figure 4). A vitality score of 6 or less was considered as failure.

### 2.6. Statistical Analysis

Statistical analyses were performed in R version 4.2.2 (R Core Team, Vienna, Austria) using the packages “moments”, “ggpubr”, “stats”, “emmeans”, and “multcompView”. The significance level was set at *p* < 0.05.

The effect of puppy weight on blood profile variables was analyzed using separate one-way ANOVA with the four puppy weight groups (quartiles) as categorical predictors. The normality of blood-profile-dependent variables was assessed through a visual inspection of histograms, Q-Q plots, and the skewness and kurtosis of data. Post hoc pairwise comparisons between quartiles were carried out using Tukey HSD tests. 

The effect of puppy weight on newborn puppy vitality scores at different time points (1 min AB, 5 min AB, 60 min AB) was evaluated using a linear mixed model with puppy vitality score set as the response variable. Puppy weight groups (quartiles) and timepoint were fitted as fixed effects, and puppy ID was set as a random effect to account for the nonindependence of puppies across time points. The normality and homoscedasticity of model residuals were assessed with a visual inspection of Q-Q plots and residuals vs. predicted values plots. Post hoc pairwise comparisons between groups and time points were conducted using Tukey HSD tests.

Differences in the proportion of stillbirths grouped according to birth weight (quartiles) and the presence of meconium stain on their skin were assessed using a chi-square test.

### 2.7. Ethical Statement 

The Ph.D. Program in Biological and Health Science Academic Committee approved the present study (CBS.114.19). The care and use of animals was performed according to guidelines for the ethical use of animals in applied ethology studies [48]. The owners provided informed consent. 

## 3. Results

A one-way ANOVA was used to determine significant differences between puppy weight quartiles in pH (F(3) = 102.70, *p* < 0.001), pCO_2_ (F(3) = 30.97, *p* < 0.001), pO_2_ (F(3) = 54.53, *p* < 0.001), glucose (F(3) = 4.581, *p* = 0.004), Ca^++^ (F(3) = 67.36, *p* < 0.001), lactate (F(3) = 48.27, *p* < 0.001), hematocrit (F(3) = 51.98, *p* < 0.001), and HCO_3_ (F(3) = 40.28, *p* < 0.001). The post hoc Tukey HSD pairwise comparisons between quartiles for the blood profile variables of puppies born alive are shown in Table 1.

Table 1 shows how some blood values in the puppies born alive presented marked variations between Q_1_ and Q_4_, such as PCO_2_, with the highest significant difference between these quartiles. If we compare the blood values obtained in Q_1_ with Q_4_, the biggest changes were reflected in Q_4_: PO_2_ decreased by 5.5 mmHg, glucose decreased by 11.8 mg/dL, hematocrit increased by 6.5%, and HCO_3_ decreased by 4.7 mmol/L. Lactate showed significant changes between Q_1_ and Q_4_, doubling its value in the quartile of puppies with the highest weight. All these findings can be assumed as indicators of stress to which larger puppies were subjected, which can be evidenced by hypercapnia, hypoxia, hypoglycemia, and marked metabolic acidosis, as well as polycythemia, hypercalcemia, and marked hyperlactatemia.

A statistically significant difference between puppy weight quartiles in the blood profile variables pCO_2_ (F(3) = 10.74, *p* < 0.001), pO_2_ (F(3) = 12.04, *p* < 0.001), lactate (F(3) = 11.88, *p* < 0.001), and HCO_3_ (F(3) = 7.316, *p* < 0.001) were found. Unlike these results, pH (F(3) = 2.217, *p* = 0.0943), glucose (F(3) = 0.177, *p* = 0.912), Ca^++^ (F(3) = 2.073, *p* = 0.112), and hematocrit (F(3) = 0.672, *p* = 0.572) did not show a significant difference between puppy weight quartiles. The post hoc Tukey HSD pairwise comparisons between quartiles for the blood profile variables of the stillbirth puppies are shown in Table 2.

Table 2 reports the blood profile alterations between the four quartiles in the SB puppies, and as shown in Table 1 for LB, many parameter alterations were observed. However, in this case, all the blood profile indicators in SB showed acid–base imbalance, manifested with a pH below 7 that denotes severe metabolic acidosis. In the same way, there was hypercapnia in the four quartiles, which was more marked in Q_4_. Also, there were marked decreases in glucose, with values ranging from 37.7 mg/dL (Q_4_) to 41 mg/dL (Q_1_), and a notable increase in Ca^++^ in all quartiles regardless of weight. In addition, lactate showed extremely high values—practically tripled if we compare them with the lactate values for LBs (LB 8.29 mmol/L and SB 14.7 mmol/L). Elevated hematocrit again denoted splenic contraction because of the release of catecholamines, and HCO_3_ was found in values decreased to half of those presented in this parameter in LBs due to the marked metabolic acidosis that the newborns experienced until death.

A linear mixed model (LMM) revealed that newborn puppies’ vitality score was significantly affected by the interaction between puppy weight group (quartile) and timepoint (F(6,722) = 5.652, *p* < 0.001). The post hoc Tukey HSD pairwise comparisons between quartiles and time after birth (AB) are reported in Table 3.

In Table 3, we can see that Q_1_ puppies were the most vigorous, and Q_4_ had the lowest scores in terms of vitality. However, after 60 min AB, the vitality began to become uniform, showing an increase in the vitality score to almost double in Q_4_ (from 5.77 min 1 AB to 7.48 min 60 AB), with statistically significant differences ceasing to exist between the four quartiles 60 min AB.

The results of the two-way ANOVAs for blood profile variables showed that the interaction between birth weight quartiles and puppy vitality score groups significantly affected the levels of pCO_2_ (F(6) = 3.389, *p* = 0.003), pO_2_ (F(6) = 3.539, *p* = 0.002), Ca^++^ (F(6) = 2.738, *p* = 0.013), lactate (F(6) = 3.091, *p* = 0.006), hematocrit (F(6) = 6.628, *p* < 0.001), and HCO_3_ (F(6) = 6.628, *p* < 0.001) in the newborn puppies. Furthermore, pH and glucose levels differed significantly between puppy weight quartiles (pH: F(3) = 184.034, *p* < 0.001; glucose: F(3) = 8.686, *p* < 0.001) and across puppies classified according to vitality score (pH: (F(2) = 140.863, *p* < 0.001; F(2) = 159.742, *p* < 0.001). The post hoc Tukey HSD pairwise comparisons between puppy weight quartiles and vitality score groups are reported in Table 4.

A statistically significant effect on vitality score for all blood values on puppies classified according to weight was observed (Table 4). Glucose and pH had no interactions, while pCO_2_, pO_2_, Ca^++^, lactate, hematocrit, and HCO_3_ had interactions and were significantly affected. Also, in all quartiles, the values that were most affected in puppies with failed vitality were pCO_2_ (increased), pO_2_ (decreased), lactate (doubled), glucose (halved), and HCO_3_ (almost halved). The results demonstrate how the puppies that presented the lowest vitality scale (failed) fought to survive, thereby causing extreme metabolic acidosis because of the activation of all the compensatory factors the newborn uses to survive.

All blood profile variables maintained a significant interaction between birth weight and the variation in the degree of meconium staining (pH: F(9) = 4.538, *p* < 0.001; pCO_2_: F(9) = 4.349, *p* < 0.001; pO_2_: F(9) = 3.287, *p* < 0.001; glucose: F(9) = 5.318, *p* < 0.001; Ca^++^: F(9) = 4.792, *p* < 0.001; lactate: F(9) = 3.751, *p* < 0.001; hematocrit: F(9) = 5.599, *p* < 0.001; and HCO_3_: F(9) = 6.013, *p* < 0.001). The post hoc Tukey HSD pairwise comparisons are shown in Table 5.

Table 5 shows that puppies with a higher degree of meconium staining presented low pH values. pCO_2_ and Ca^++^ increased in severe meconium staining, and pO_2_ decreased. These findings suggest that as the degree of meconium staining increased, more significant blood profile alterations were observed regardless of weight or quartile. Ca^++^, pCO2, hematocrit, and lactate values increased directly in severe meconium staining. On the contrary, in severe meconium staining, the pH, pO_2_, glucose, and HCO_3_ decreases were inversely proportional. 

A chi-square test was performed to determine whether the proportion of stillbirths differed between puppies classified according to birth weight groups and the presence of meconium staining. The proportion of stillbirth puppies significantly differed between puppy weight quartiles and degrees of meconium staining (χ^2^ = 89.475, df = 15, *p* < 0.001). The number and percentage of liveborn (LB) and stillbirths (SBs) can be observed in Table 6.

Table 6 shows that regardless of weight or quartile, the degree of meconium staining significantly affected the number of SBs, which was greater in severe meconium staining and lower or even null in absent meconium staining degree.

The correlation coefficients for the blood profile values, the degree of meconium staining, and the vitality score are reported in Table 7, Table 8, and Table 9, respectively.

Table 7 shows that in Q_1,_ weight significantly affected pCO2, with a positive correlation of 0.273 (*p* = 0.004). In Q_2,_ there was a significant effect of weight on pCO_2_, lactate, glucose, hematocrit, and HCO_3_. Q_3_ had a positive correlation with glucose (*p* < 0.001) and calcium (*p* = 0.027). In the case of Q_4_, there were significantly high positive and negative correlations, for all the variables with statistically significant levels of *p* < 0.001, so it can be concluded that having more weight causes high positive correlations with all blood variables. We have marked with an asterisk the values in which there were statistically significant correlations.

Table 8 reports that birth weight was correlated with the degree of meconium staining in Q_2_, with *r*^2^ = −0.200 and *p* = 0.038, and in Q_4_, with *r*^2^ = 0.214 and *p* = 0.025, so it can be stated that birth weight was correlated with meconium staining in Q_2_ and Q_4_. We have marked with an asterisk the values in which there were statistically significant correlations.

Table 9 shows that no statistically significant correlations were found in any of the groups or at any time due to the effect of birth weight and vitality.

## 4. Discussion

The results show that birth weight influences the thermoregulation, hemodynamic changes, and degree of meconium staining in newborn dogs, as reported in previous studies performed by the authors [4,49].

### 4.1. Blood Profile Values 

The most notable blood profile changes were associations between Q_4_ puppies (heavier newborns) with hypoxia, hypoglycemia, hypercalcemia, hyperlactatemia, and marked metabolic acidosis.

The plasma levels of HCO_3_^−^ observed in this study were 21.7 mmol/L for Q_1_ LB, 12.8 mmol/L for Q_1_ SBs, 17 mmol/L for Q_4_ LB, and 10.6 mmol/L for Q_4_ SB. These values could demonstrate severe pulmonary hypoxia and elevated lactic acid production because when its production is greatly increased, the organism activates the use of HCO_3_ as a buffer. However, because of the increased consumption of HCO_3_^−^, newborns experience mixed (respiratory and metabolic) acidosis and metabolic deficiencies due to these anaerobic and respiratory processes that ultimately lead to hypoxia [50].

Variations in the balance of glucose levels during the neonatal stage depend on endogenous production and glucose metabolism, resulting in hyper- or hypoglycemia. According to Vannucchi et al. [51], hypoglycemia is the leading cause of neonatal mortality because a rapid depletion of energy resources can cause acute hypothermia. In other words, the rapid reduction in fetal circulation at delivery caused by uterine contractions and the pressure they can exert on the fetal umbilical cord is associated with rapid liver glycogen depletion and decreased glucose homeostasis [52]. In our study, within these adverse circumstances, we can mention that for Q_4,_ the whelping usually took longer because they are breeds that give birth to litters that are larger in number and size, which can prolong the expulsion interval. The significant differences in blood glucose concentration in the quartiles (62.8 mg/dL in Q_1_; 65.3 mg/dL in Q_2_; 71.3 mg/dL in Q_3,_ and 62.6 mg/dL in Q_4_) could be associated with neonatal stress and the consequent release of catecholamine and promotion of liver glycogenolysis [53,54].

The reduction in the oxygen supply observed in the present study was also reported by Mota-Rojas et al. [46,47] in piglets, in which umbilical cord hypoxia leads to increased pCO_2_ due to respiratory acidosis, acting as a risk for prenatal mortality. Similarly, in another study by Massip [16] in fetal sheep near term, the animals showed a compensatory mechanism to respiratory acidosis by increasing pCO_2._ The contrary is observed during metabolic acidosis, leading to alveolar hyperventilation secondary to increased H^+^ ions in the plasma and a decrease in pCO_2_.

These mentioned findings coincide with what was found in our study, where once again, the quartile of the heaviest puppies (Q_4_) developed severe hypercapnia, ranging from 64 mmHg in LB to 95.1 mmHg in SB, and this could be explained by the expectation that during birth, there may be variations in cardiorespiratory parameters. Tachycardia, hypercapnia, and respiratory acidosis may arise immediately after birth. According to Alonso-Spilsbury et al. [55] and Vannucchi et al. [51], physiological hypoxia triggers an increase in respiratory rate and its consequent apnea. The important variations in pH in all quartiles, especially in Q_4_ (LB 7.24; SB 6.82), show that bicarbonate and pCO_2_ indicate neonatal acidosis and possible fetal hypoxic stress [56].

### 4.2. Vitality Score

Puppies of Q_4_ had the lowest vitality rating of all the quartiles at minute 1 AB. This could be associated with some factors, such as the expulsion interval, since in smaller puppies, it is faster than in larger ones. Therefore, unlike larger puppies, which take longer to be expelled, the smaller ones do not consume energy reserves as much. Hence, the energy consumption in larger puppies to try to compensate for the stress of hypoxia is more significant in the expulsion phase. These biggest newborns have an increased risk of intrauterine asphyxia, affecting their survival chances [26]. 

Birth weight can also predispose to asphyxia. For example, in humans, low-birth- weight individuals have poor vitality scores [14]. Similarly, Okere et al. [57] found that piglet viability score was highly correlated with their weight (*r* = 0.66). In contrast to these studies, we found that the biggest puppies (Q_4_) had low vitality scores at the first minute AB, but their score improved at minute 60 AB. Our results are similar to the ones reported by Trujillo et al. [58], who concluded that heavy piglets had scores ≤ 5 and showed asphyxia signs. A possible explanation could be that heavier piglets had greater difficulty passing through the birth canal, affecting the health and vitality of the newborns [4,59]. Additionally, Veronesi et al. [60] mention that small-sized puppies may have the highest levels of distress but higher chances of survival when compared to large-sized animals.

Vitality also influences the capability of a newborn to stand and ingest colostrum, limiting the absorption of nutrients and energy obtention [61], parameters that are important in the postpartum period because the disposition of energy resources, such as brown adipose tissue and glucose, differs between low- and high-birth-weight animals. Moreover, blood oxygenation, particularly oxygen saturation (SpO2), has shown a negative correlation with birth weight, meaning that heavier newborns have lower SpO2, and might influence the time to receive oxygen [62]. 

Surprisingly, as the minutes passed, it was possible to observe that the time factor favored Q_4_ because the consumption of colostrum probably matched these puppies, and because at minute 60 AB, almost always, all newborns have already consumed colostrum, in addition to the fact that larger puppies will have more suction force and, therefore, consume more colostrum. This could be sustained with a study by Mila et al. [42], who mentioned that glucose in the colostrum is essential to newborn puppies because only 1.3% of the body fat is available. On the contrary, the colostrum of small-breed bitches (less than 10 kg) provides 10% more energy than the colostrum of large-breed females [3].

In the same way that Herpin et al. [53] and Trujillo-Ortega et al. [58,63] reported, newborn puppies that failed the vitality scale in our study showed a blood rise in pCO_2_ and lactic acid, with a low pO_2_ in plasma, finding that would confirm that these newborns suffered early tissue hypoxia.

Correlations between birth weight and vitality did not show a statistically significant difference, which can be explained by the fact that, even though the puppies in Q_4_ showed failed vitality scores (5.77) at minute 1 AB, they managed to reach high vitality scores (7.48) at minute 60 AB, practically matching the vitality scores of the puppies of the other quartiles (Q_1_, Q_2_, and Q_3_).

### 4.3. Meconium Staining Degree

Studies performed on piglets suggest that low-weight newborns have a higher frequency of intrapartum mortality [38,64,65]. In contrast, human babies with higher birth weights appear to have a higher predisposition to hypoxia and meconium aspiration [66,67]. This association could be related to parturition problems due to the size and weight of the newborn rather than problems with the umbilical cord. This also could explain why heavier intrapartum piglets showed more severe meconium staining in a study by Mota-Rojas et al. [30].

The present results show that 16.5% of stillbirths had severe meconium staining of the skin in Q_4_ puppies. Most stillbirths had mild, moderate, and severe meconium staining (6, 10, and 18 puppies, respectively), suggesting that careful observation is required to assess intrapartum anoxia based on epidermal staining alone correctly. In other words, the highest percentages of stillbirths occurred in Q_4,_ with 33.02% (36 SB puppies from 109 in total), of which 5.5% were mild meconium staining, 9.1% were moderate, and 16.5% were severe. 

The present study found an association between low blood pH values and severe meconium staining, regardless of birthweight, coinciding with previous studies where meconium staining demonstrated the ability to lead to fetal hypoxia and, consequently, acidosis [68]. The presence of meconium can obstruct the airways, leading to acute hypoxemia, hypercapnia, and metabolic acidosis due to tissular anaerobic metabolism that causes a progressive decrease in pulmonary blood flow, exacerbating hypoxia and acidosis [69]. A similar result was reported in humans, in whom 81.82% of newborns that presented thick meconium-stained fluid and low Apgar scores (around 6.45) had blood pH below 7.5 [70]. Therefore, observing meconium-stained puppies could indicate physiological alterations that might not always be evaluated during whelping but could be inferred to adopt a proper perinatal control of stained puppies.

One of the main limitations of this study was that the monitoring of the animals was carried out in 10 different clinics or hospitals to have a larger number of samples. For this reason, factors such as standardizing the temperatures where the births took place were not possible, but to standardize the areas where the dams gave birth, foam mats and adult bed cover diapers were used. However, parturitions were carried out in Campeche, Mexico, where there is a tropical climate, so situations such as hypothermia due to environmental factors were not observed. As it is a species where the breeds have a great diversity of weights and sizes, the authors decided to classify the animals according to their weight and not their breed. Therefore, another limitation could be that some breeds could have more intense maternal care than others or have longer parturitions due to the size of the litter or newborns.

## 5. Conclusions

In the present study, we could observe that the weight of newborns was correlated with the degree of meconium staining, presenting more cases of severe meconium staining in the puppies of the highest-birth-weight group. In addition, the weight of the newborns was correlated with the alterations in the blood variables, showing the most severe cases of metabolic acidosis, hypoxia, hypoglycemia, and higher stillbirths in the puppies of the Q_4_ quartile. On the contrary, no statistically significant correlations were found between the weight of newborns and vitality. However, it could be observed throughout the analysis of the results that the most vigorous puppies were found in Q_1._ However, at minute 60 AB, all the puppies in the four quartiles standardized their vitality scores. According to this, our hypothesis that bigger newborns would have lower scores in vitality, more blood profile alterations, and more cases of meconium staining than smaller puppies was confirmed.

One of the main perspectives that we were able to elucidate with this study, and according to Groppetti et al. [71] and Forsberg [72], is that the use of both fetal and uterine monitoring before, during, and after parturition could be a valuable tool to help predict cases of hypoxia, asphyxia, dystocia, and uterine inertia, which could lead to prolonged deliveries with complications and, therefore, an increase in the mortality rate as well as a decrease in the vitality of newborns [73,74,75,76].

## Figures and Tables

**Table 1:** Mean and standard error of blood profile values from puppies born alive and classified according to their birth weight.

Blood Trait	Q_1_ *n =* 110	Q_2_ *n =* 108	Q_3_ *n =* 108	Q_4_ *n =* 109
pH	7.49 ± 0.0126 ^a^	7.24 ± 0.0136 ^b^	7.25 ± 0.0116 ^b^	7.24 ± 0.0125 ^b^
pCO_2_ (mmHg)	46.9 ± 0.879 ^a^	56.5 ± 1.41 ^b^	58.8 ± 1.46 ^b^	64.0 ± 1.42 ^c^
pO_2_ (mmHg)	18.0 ± 0.325 ^a^	14.4 ± 0.323 ^b^	14.0 ± 0.302 ^b^	12.5 ± 0.306 ^c^
Glucose (mg/dL)	93.7 ± 1.83 ^a^	92.7 ± 2.44 ^a^	93.4 ± 2.76 ^a^	81.9 ± 2.99 ^b^
Ca^++^ (mmol/L)	1.59 ± 0.0196 ^a^	1.90 ± 0.0206 ^a^	1.90 ± 0.0200 ^a^	1.94 ± 0.0208 ^b^
Lactate (mmol/L)	4.74 ± 0.226 ^a^	6.86 ± 0.198 ^a,b^	7.54 ± 0.216 ^b^	8.29 ± 0.252 ^c^
Hematocrit (%)	45.3 ± 0.408 ^a^	48.7 ± 0.348 ^b^	50.0 ± 0.404 ^b^	51.8 ± 0.316 ^c^
HCO_3_^−^ (mmol/L)	21.7 ± 0.357 ^a^	19.0 ± 0.322 ^b^	17.8 ± 0.305 ^b,c^	17.0 ± 0.321 ^c^

(One-way ANOVA.) Q_1_, 127–200 g; Q_2_, 201 g–269 g; Q_3_, 270 g–388 g; Q_4_, 389 g–464 g. ^a,b,c^ Different superscripts between columns indicate statistically significant differences between quartiles with *p* < 0.05.

**Table 2:** Mean and standard error of blood profile values in stillbirth puppies classified according to their birth weight.

Blood Trait	Q_1_ *n =* 100	Q_2_ *n =* 108	Q_3_ *n =* 108	Q_4_ *n =* 109
pH	6.82 ± 0.0479 ^a^	6.80 ± 0.0449 ^a^	6.73 ± 0.0325 ^a^	6.82 ± 0.0176 ^a^
pCO_2_ (mmHg)	82.5 ± 2.37 ^a^	83.2 ± 1.34 ^ab^	91.7 ± 1.48 ^c^	95.1 ± 1.36 ^b,c^
pO_2_ (mmHg)	9.24 ± 0.531 ^a^	8.2 ± 0.932 ^a^	5.71 ± 0.486 ^b^	4.34 ± 0.386 ^b^
Glucose (mg/dL)	41.0 ± 6.06 ^a^	40.0 ± 4.34 ^a^	37.7 ± 2.10 ^a^	37.7 ± 2.26 ^a^
Ca^++^ (mmol/L)	2.12 ± 0.0630 ^a^	2.12 ± 0.0650 ^a^	2.26 ± 0.0474 ^a^	2.17 ± 0.0216 ^a^
Lactate (mmol/L)	12.3 ± 0.682 ^a^	12.1 ± 0.480 ^a^	14.3 ± 0.340 ^b^	14.7 ± 0.226 ^b^
Hematocrit (%)	59.9 ± 1.01 ^a^	60.5 ± 0.423 ^a^	59.3 ± 0.725 ^a^	59.1 ± 0.585 ^a^
HCO_3_^−^ (mmol/L)	12.8 ± 0.685 ^a^	12.8 ± 0.685 ^a^	13.2 ± 0.571 ^a,b^	10.6 ± 0.343 ^b^

(One-way ANOVA.) Q_1_, 127–200 g; Q_2_, 201 g–269 g; Q_3_, 270 g–388 g; Q_4_, 389 g–464 g. ^a,b,c^ Different superscripts between columns indicate statistically significant differences between quartiles with *p* < 0.05.

**Table 3:** Estimated marginal mean and standard error of vitality score of newborn puppies evaluated at different times and classified according to their birth weight.

Time	Q_1_ *n =* 110	Q_2_ *n =* 108	Q_3_ *n =* 108	Q_4_ *n =* 109
Minute 1 AB	7.28 ± 0.173 ^a,1^	6.80 ± 0.180 ^a,1^	6.62 ± 0.187 ^a,b,1^	5.77 ± 0.207 ^b,1^
Minute 5 AB	7.64 ± 0.173 ^a,1,2^	7.38 ± 0.180 ^a,2^	7.28 ± 0.187 ^a,2^	6.81 ± 0.207 ^a,2^
Minute 60 AB	7.95 ± 0.173 ^a,2^	7.73 ± 0.180 ^a,2^	7.68 ± 0.187 ^a,2^	7.48 ± 0.207 ^a,3^

(Linear mixed model.) Q_1_, 127–200 g; Q_2_, 201 g–269 g; Q_3_, 270 g–388 g; Q_4_, 389 g–464 g. AB: After birth. ^a,b^ Different superscripts between columns indicate statistically significant differences between quartiles with *p* < 0.05. ^1,2,3^ Different numbers among rows indicate statistically significant differences between times of evaluation.

**Table 4:** Mean and standard error of blood profile values in newborn puppies born alive classified according to their vitality score (failed, medium, and high) and birth weight.

Blood Trait	V 0–5 (Failed) *n* = 102	V 6–7 (Medium) *n* = 105	V 8–10 (High) *n* = 158
Q_1_			
pH	7.29 ± 0.0207 ^a,1^	7.50 ± 0.0197 ^b,1^	7.55 ± 0.0108 ^b,1^
pCO_2_ (mmHg)	62.3 ± 2.30 ^a,1^	42.5 ± 1.07 ^b,1^	43.8 ± 0.362 ^b,1^
pO_2_ (mmHg)	13.7 ± 0.578 ^a,1^	18.7 ± 0.697 ^b,1^	19.0 ± 0.306 ^b,1^
Glucose (mg/dL)	62.8 ± 4.73 ^a,1^	99.4 ± 2.55 ^b,1^	101.0 ± 0.943 ^b,1^
Ca^++^ (mmol/L)	1.92 ± 0.0519 ^a,1^	1.56 ± 0.0259 ^b,1^	1.51 ± 0.0108 ^b,1^
Lactate (mmol/L)	8.09 ± 0.639 ^a,1^	4.25 ± 0.325 ^b,1^	3.91 ± 0.163 ^b,1^
Hematocrit (%)	52.2 ± 0.858 ^a,1^	43.6 ± 0.555 ^b,1^	43.8 ± 0.273 ^b,1^
HCO_3_^−^ (mmol/L)	15.1 ± 0.665 ^a,1^	22.2 ± 0.328 ^b,1^	23.6 ± 0.189 ^b,1^
Q_2_			
pH	7.09 ± 0.0214 ^a,2^	7.27 ± 0.0159 ^b,2^	7.31 ± 0.0196 ^b,2^
pCO_2_ (mmHg)	75.3 ± 2.83 ^a,2^	53.9 ± 1.84 ^b,2^	48.6 ± 0.447 ^b,1,2^
pO_2_ (mmHg)	11.0 ± 0.502 ^a,2^	14.4 ± 0.443 ^b,2^	16.4 ± 0.370 ^c,2^
Glucose (mg/dL)	65.3 ± 4.67 ^a,1^	95.1 ± 3.21 ^b,1^	106.0 ± 2.59 ^b,1^
Ca^++^ (mmol/L)	2.08 ± 0.0500 ^a,1^	1.87 ± 0.0294 ^b,2^	1.82 ± 0.0231 ^b,2^
Lactate (mmol/L)	9.08 ± 0.416 ^a,1,2^	6.96 ± 0.249 ^b,2^	5.50 ± 0.145 ^c,2^
Hematocrit (%)	51.5 ± 0.871 ^a,1^	47.9 ± 0.519 ^b,2^	47.8 ± 0.380 ^b,2^
HCO_3_^−^ (mmol/L)	14.6 ± 0.608 ^a,1^	19.8 ± 0.385 ^b,2^	20.6 ± 0.213 ^b,2^
Q_3_			
pH	7.14 ± 0.0183 ^a,1,2^	7.26 ± 0.0164 ^b,2^	7.32 ± 0.0122 ^b,2^
pCO_2_ (mmHg)	71.0 ± 2.70 ^a,1^	57.1 ± 2.65 ^b,2^	52.1 ± 1.28 ^b,2^
pO_2_ (mmHg)	11.3 ± 0.472 ^a,1,2^	14.2 ± 0.429 ^b,2^	15.6 ± 0.371 ^b,2^
Glucose (mg/dL)	71.3 ± 5.24 ^a,1^	92.7 ± 4.69 ^b,1^	108.0 ± 2.44 ^c,1^
Ca^++^ (mmol/L)	2.05 ± 0.0427 ^a,1,2^	1.89 ± 0.0336 ^b,2^	1.81 ± 0.0171 ^b,2^
Lactate (mmol/L)	9.75 ± 0.226 ^a,2^	7.16 ± 0.377 ^b,2^	6.36 ± 0.212 ^b,2^
Hematocrit (%)	52.2 ± 0.976 ^a,1^	48.9 ± 0.538 ^b,2^	49.2 ± 0.498 ^b,2,3^
HCO_3_^−^ (mmol/L)	14.4 ± 0.382 ^a,1^	18.6 ± 0.505 ^b,2,3^	19.5 ± 0.204 ^b,2^
Q_4_			
pH	7.17 ± 0.0150 ^a,2^	7.28 ± 0.0147 ^b,2^	7.33 ± 0.0169 ^b,2^
pCO_2_ (mmHg)	71.4 ± 1.49 ^a,2^	61.8 ± 3.13 ^b,2^	51.9 ± 0.782 ^b,2^
pO_2_ (mmHg)	11.2 ± 0.395 ^a,2^	14.4 ± 0.507 ^b,2^	13.3 ± 0.471 ^a,b,3^
Glucose (mg/dL)	62.6 ± 1.82 ^a,1^	89.8 ± 6.21 ^b,1^	111.0 ± 1.96 ^c,1^
Ca^++^ (mmol/L)	2.06 ± 0.0178 ^a,2^	1.90 ± 0.0448 ^b,2^	1.76 ± 0.0193 ^b,2^
Lactate (mmol/L)	9.68 ± 0.228 ^a,2^	8.26 ± 0.428 ^a,2^	5.75 ± 0.226 ^b,2^
Hematocrit (%)	53.1 ± 0.369 ^a,1^	50.1 ± 0.546 ^b,2^	50.6 ± 0.593 ^a,b,3^
HCO_3_^−^ (mmol/L)	15.4 ± 0.349 ^a,1^	17.2 ± 0.690 ^a,3^	19.7 ± 0.230 ^b,2^

(Two-way ANOVA.) Q_1_, 127–200 g; Q_2_, 201 g–269 g; Q_3_, 270 g–388 g; Q_4_, 389 g–464 g. V: Vitality. ^a,b,c^ Different superscripts between columns indicate statistically significant differences between vitality score and quartiles with *p* < 0.05. ^1,2,3^ Different numbers among rows indicate statistically significant differences between blood values on different quartiles.

**Table 5:** Effect of the meconium staining degree on blood profile values of newborn puppies classified according to birth weight.

Blood Trait	Absent	Mild	Moderate	Severe
Q_1_				
pH	7.54 ± 0.0098 ^a,1^	7.47 ± 0.0323 ^a,1^	7.11 ± 0.0919 ^b,1^	7.21 ± 0.0476 ^b,1^
pCO_2_ (mmHg)	43.5 ± 0.378 ^a,1^	45.4 ± 2.45 ^a,1^	69.8 ± 2.45 ^b,1^	67.9 ± 3.79 ^b,1^
pO_2_ (mmHg)	18.9 ± 0.314 ^a,1^	18.5 ± 0.666 ^a,1^	11.8 ± 0.869 ^b,1^	12.2 ± 0.482 ^b,1^
Glucose (mg/dL)	101.00 ± 0.877 ^a,1^	95.4 ± 4.70 ^a,1^	55.0 ± 5.59 ^b,1^	51.0 ± 3.76 ^b,1^
Ca^++^ (mmol/L)	1.51 ± 0.0102 ^a,1^	1.60 ± 0.0485 ^a,1^	2.04 ± 0.0371 ^b,1^	1.98 ± 0.0659 ^b,1^
Lactate (mmol/L)	3.87 ± 0.145 ^a,1^	4.96 ± 0.604 ^a,1^	8.95 ± 0.736 ^b,1,2^	10.1 ± 0.749 ^b,1^
Hematocrit (%)	43.8 ± 0.259 ^a,1^	44.9 ± 1.49 ^a,1^	55.0 ± 1.21 ^b,1^	54.0 ± 1.15 ^b,1^
HCO_3_^−^ (mmol/L)	23.3 ± 0.189 ^a,1^	21.9 ± 0.468 ^a,1^	14.1 ± 0.435 ^b,1^	13.5 ± 0.402 ^b,1^
Q_2_				
pH	7.29 ± 0.0154 ^a,2^	7.26 ± 0.0224 ^a,1,2^	7.14 ± 0.0280 ^a,1^	6.90 ± 0.0380 ^b,2^
pCO_2_ (mmHg)	49.6 ± 0.774 ^a,1,2^	60.5 ± 3.56 ^a,b,1,2^	68.2 ± 4.42 ^b,1^	82.6 ± 1.31 ^c,2^
pO_2_ (mmHg)	15.5 ± 0.371 ^a,2^	13.8 ± 0.824 ^a,2^	12.5 ± 0.484 ^a,b,1^	9.04 ± 0.637 ^b,1,2^
Glucose (mg/dL)	103.0 ± 1.99 ^a,1^	87.6 ± 5.28 ^a,b,1^	76.0 ± 6.78 ^b,1^	44.3 ± 3.75 ^c,1^
Ca^++^ (mmol/L)	1.82 ± 0.0176 ^a,2^	1.95 ± 0.0453 ^a,b,2^	1.99 ± 0.0560 ^b,1^	2.15 ± 0.0595 ^b,1^
Lactate (mmol/L)	6.02 ± 0.184 ^a,2^	7.74 ± 0.373 ^a,1,2^	8.18 ± 0.596 ^a,,1^	11.0 ± 0.470 ^b,1^
Hematocrit (%)	48.2 ± 0.435 ^a,2^	49.0 ± 1.20 ^a,1,2^	52.3 ± 1.14 ^a,b,1^	54.2 ± 1.44 ^b,1^
HCO_3_^−^ (mmol/L)	20.6 ± 0.247 ^a,2^	19.2 ± 0.469 ^a,1,2^	14.8 ± 0.562 ^b,1^	13.1 ± 0.380 ^b,1^
Q_3_				
pH	7.28 ± 0.0185 ^a,2^	7.14 ± 0.0614 ^a,b,2,3^	7.05 ± 0.0704 ^b,1^	7.00 ± 0.0409 ^b,2^
pCO_2_ (mmHg)	53.9 ± 1.58 ^a,2^	64.9 ± 5.03 ^a,b,2^	77.2 ± 4.34 ^b,1^	77.7 ± 2.92 ^b,1,2^
pO_2_ (mmHg)	14.9 ± 0.405 ^a,2,3^	12.8 ± 0.976 ^a,b,2^	9.75 ± 1.01 ^b,1^	9.42 ± 0.709 ^b,1,2^
Glucose (mg/dL)	105.0 ± 2.79 ^a,1^	74.9 ± 7.15 ^b,1,2^	63.4 ± 8.55 ^b,1^	58.7 ± 4.92 ^b,1^
Ca^++^ (mmol/L)	1.83 ± 0.0219 ^a,2^	1.98 ± 0.0630 ^a,b,2^	2.10 ± 0.0688 ^b,1^	2.12 ± 0.0378 ^b,1^
Lactate (mmol/L)	6.51 ± 0.258 ^a,2^	9.46 ± 0.899 ^b,2,3^	10.8 ± 0.832 ^b,1,2^	11.4 ± 0.399 ^b,1^
Hematocrit (%)	49.7 ± 0.445 ^a,2^	51.2 ± 1.60 ^a,b,2,3^	53.6 ± 1.89 ^a,b,1^	54.2 ± 0.941 ^b,1^
HCO_3_^−^ (mmol/L)	19.5 ± 0.207 ^a,2^	17.8 ± 0.712 ^a,2,3^	14.2 ± 0.526 ^b,1^	13.2 ± 0.273 ^b,1^
Q_4_				
pH	7.29 ± 0.0275 ^a,2^	7.00 ± 0.0670 ^b,3^	7.08 ± 0.0445 ^b,1^	7.02 ± 0.0283 ^b,2^
pCO_2_ (mmHg)	55.3 ± 2.13 ^a,2^	83.5 ± 3.65 ^b,3^	76.2 ± 3.05 ^b,1^	83.1 ± 2.43 ^b,2^
pO_2_ (mmHg)	12.7 ± 0.614 ^a,3^	8.12 ± 1.13 ^b,3^	10.0 ± 0.854 ^a,b,1^	8.10 ± 0.731 ^b,2^
Glucose (mg/dL)	103.0 ± 4.41 ^a,1^	49.8 ± 4.14 ^b,2^	61.2 ± 4.79 ^b,1^	51.9 ± 3.05 ^b,1^
Ca^++^ (mmol/L)	1.78 ± 0.0218 ^a,2^	2.08 ± 0.0288 ^b,2^	2.07 ± 0.0320 ^b,1^	2.13 ± 0.0212 ^b,1^
Lactate (mmol/L)	6.75 ± 0.443 ^a,2^	11.9 ± 0.880 ^b,3^	11.1 ± 0.630 ^b,2^	12.0 ± 0.436 ^b,1^
Hematocrit (%)	51.3 ± 0.689 ^a,2^	56.7 ± 1.50 ^b,3^	54.8 ± 0.970 ^a,b,1^	55.0 ± 0.558 ^b,1^
HCO_3_^−^ (mmol/L)	19.1 ± 0.422 ^a,2^	15.6 ± 0.970 ^b,3^	12.8 ± 0.565 ^c,1^	13.2 ± 0.514 ^b,c,1^

(Two-way ANOVA.) Q_1_, 127–200 g; Q_2_, 201 g–269 g; Q_3_, 270 g–388 g; Q_4_, 389 g–464 g. ^a,b,c^ Different superscripts between columns indicate statistically significant differences between the degree of meconium staining and quartiles with *p* < 0.05. ^1,2,3^ Different numbers among rows indicate statistically significant differences between blood values on different quartiles.

**Table 6:** Number and percentage of liveborn (LB) and stillbirth (SB) puppies according to their weight and the presence of meconium staining on the skin: absent, mild, moderate, and severe, and significant statistical differences.

Staining Degree	Q_1_ *n =* 110		Q_2_ *n* = 108		Q_3_ *n* = 108		Q_4_ *n* = 109	
	LB (%)	SB (%)	LB (%)	SB (%)	LB (%)	SB (%)	LB (%)	SB (%)
Absent	77 (70) ^a,1^	0 ^a,1^	58 (53) ^a,1^	3 (2.7) ^a,1^	51 (47.2) ^a,1^	2 (1.8) ^a,1^	26 (23.8) ^a,1^	2 (1.8) ^a,1^
Mild	12 (10.9) ^a,1,2^	0 ^a,1,2^	14 (12.9) ^a,1^	0 ^a,1^	11 (10.1) ^a,1^	3 (2.7) ^a,1^	6 (5.5) ^a,1^	6 (5.5) ^a,1^
Moderate	6 (5.4) ^a,2^	3 (2.7) ^a,2^	12 (11.1) ^a,1^	2 (1.8) ^a,1^	10 (9.2) ^a,1^	5 (4.6) ^a,1^	18 (16.5) ^a,1^	10 (9.1) ^a,1^
Severe	10 (9) ^a,1,2^	2 (1.8) ^a,1,2^	12 (11.1) ^a,1^	7 (6.4) ^a,1^	18 (16.6) ^a,1^	8 (7.4) ^a,1^	23 (21.1) ^a,1^	18 (16.5) ^a,1^

(*X*^2^ test). Q_1_, 127–200 g; Q_2_, 201 g–269 g; Q_3_, 270 g–388 g; Q_4_, 389 g–464 g. LB: liveborn; SB: stillbirth. ^a^ Different superscript between columns indicates statistically significant differences between quartiles and meconium staining degree with *p* < 0.05. ^1,2^ Different numbers among rows indicate statistically significant differences between the degree of meconium staining in liveborn and stillbirth puppies.

**Table 7:** Significant correlations between puppies’ weight and blood profile values.

Variables	Correlation Coefficient (*rho*)	*p*-Value
Q_1_		
pH	−0.125	0.193
pCO_2_ (mmHg)	0.273	0.004 *
pO_2_ (mmHg)	−0.137	0.154
Glucose (mg/dL)	−0.165	0.085
Ca^++^ (mmol/L)	0.197	0.039
Lactate (mmol/L)	0.138	0.151
Hematocrit (%)	0.119	0.217
HCO_3_^−^ (mmol/L)	−0.109	0.257
Q_2_		
pH	0.178	0.065
pCO_2_ (mmHg)	−0.205	0.034 *
PO_2_ (mmHg)	0.168	0.082
Glucose (mg/dL)	0.324	<0.001 *
Ca^++^ (mmol/L)	−0.038	0.695
Lactate (mmol/L)	−0.214	0.026 *
Hematocrit (%)	−0.266	0.005 *
HCO_3_^−^ (mmol/L)	0.224	0.019 *
Q_3_		
pH	0.167	0.084
pCO_2_ (mmHg)	−0.006	0.954
pO_2_ (mmHg)	0.012	0.900
Glucose (mg/dL)	0.304	0.001 *
Ca^++^ (mmol/L)	−0.213	0.027 *
Lactate (mmol/L)	0.063	0.516
Hematocrit (%)	0.017	0.861
HCO_3_^−^ (mmol/L)	0.039	0.686
Q_4_		
pH	−0.587	<0.001 *
pCO_2_ (mmHg)	0.621	<0.001 *
PO_2_ (mmHg)	−0.512	<0.001 *
Glucose (mg/dL)	−0.579	<0.001 *
Ca^++^ (mmol/L)	0.412	<0.001 *
Lactate (mmol/L)	0.603	<0.001 *
Hematocrit (%)	0.532	<0.001 *
HCO_3_^−^ (mmol/L)	−0.541	<0.001 *

Spearman’s rank correlation coefficients and their statistical significance between puppies’ weight and their blood profile values. * Indicates significant statistical differences.

**Table 8:** Significant correlations between puppies’ weight and meconium staining degree. The meconium staining degree has been recoded to numerical values. Higher the stain degree = higher number. Absent = 1, mild = 2, moderate = 3, and severe = 4.

Variables	Correlation Coefficient (*r*)	*p*-Value
All puppies (quartiles)	0.3319875	*p* < 0.001
Q_1_	0.111	0.248
Q_2_	−0.200	0.038 *
Q_3_	0.023	0.811
Q_4_	0.214	0.025 *

Spearman’s rank correlation coefficients and their statistical significance between puppies’ weight and their meconium staining degree. * Indicates significant statistical differences.

**Table 9:** Significant correlations between puppies’ weight and its vitality.

Variables	Correlation Coefficient (*r*)	*p*-Value
Q_1_		
Vitality Score min 1	0.0644	0.514
Vitality Score min 5	0.116	0.238
Vitality Score min 60	0.0325	0.742
Q_2_		
Vitality Score min 1	0.0587	0.568
Vitality Score min 5	0.148	0.148
Vitality Score min 60	0.174	0.0882
Q_3_		
Vitality Score min 1	−0.0323	0.762
Vitality Score min 5	0.0430	0.687
Vitality Score min 60	0.143	0.179
Q_4_		
Vitality Score min 1	0.0912	0.443
Vitality Score min 5	0.0822	0.489
Vitality Score min 60	0.0887	0.456

Spearman’s rank correlation coefficients and their statistical significance between puppies’ weight and their vitality.

## Data Availability

Not applicable.
